# Synthesis of C_20–38_ Fatty Acids in Plant Tissues

**DOI:** 10.3390/ijms23094731

**Published:** 2022-04-25

**Authors:** Anatoly Zhukov, Valery Popov

**Affiliations:** Timiryazev Institute of Plant Physiology, Russian Academy of Sciences, Botanicheskaya Street 35, 127276 Moscow, Russia; zhukov_anatoly@list.ru

**Keywords:** polar lipids, storage lipids, very-long-chain fatty acids, elongase systems, desaturation of fatty acids, cuticular waxes, suberin

## Abstract

Very-long-chain fatty acids (VLCFA) are involved in a number of important plant physiological functions. Disorders in the expression of genes involved in the synthesis of VLCFA lead to a number of phenotypic consequences, ranging from growth retardation to the death of embryos. The elongation of VLCFA in the endoplasmic reticulum (ER) is carried out by multiple elongase complexes with different substrate specificities and adapted to the synthesis of a number of products required for a number of metabolic pathways. The information about the enzymes involved in the synthesis of VLCFA with more than 26 atoms of Carbon is rather poor. Recently, genes encoding enzymes involved in the synthesis of both regular-length fatty acids and VLCFA have been discovered and investigated. Polyunsaturated VLCFA in plants are formed mainly by 20:1 elongation into new monounsaturated acids, which are then imported into chloroplasts, where they are further desaturated. The formation of saturated VLCFA and their further transformation into a number of aliphatic compounds included in cuticular waxes and suberin require the coordinated activity of a large number of different enzymes.

## 1. Introduction

**Figure 1:**
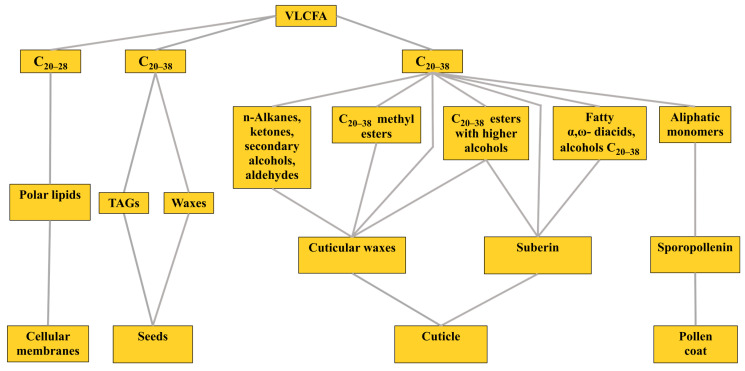
The distribution of very-long-chain fatty acids (VLCFA) in plant cells and tissues. C_20–28_ VLCFA are the components of polar lipids (PL) of cell membranes. C_20–38_ VLCFA are found in developing seeds as a part of reserve triacylglycerols and waxes and are also included in cuticular waxes, suberin and sporopollenin, which form a surface barrier on leaves, stems, roots and pollen shells, respectively.

Very-long-chain fatty acids (VLCFA) are molecules with a hydrocarbon chain containing 20–38 atoms of C [1], including an even, and sometimes an odd, number of these atoms and are capable of converting into hydroxy-FA. In this paper, we mainly consider only the even ones from C_20–38_ VLCFA, and the odd ones are mentioned separately. C_20–38_ VLCFA are involved in various physiological processes in plants. They are components of polar lipids of biomembranes that, together with other compounds, create a surface barrier on leaves, stems and roots and are also part of the storage lipids in the seeds of some plant species (Figure 1) [2,3,4,5,6,7]. An important role of VLCFA is their participation in the construction of any cell membranes of higher plants and algae. Their number in plasma membranes can be 5–10% of the amount of fatty acids (FA) [2]. C_20–28_ VLCFA are components of biomembranes in the composition of phospholipids (PhL), glycero- and sphingo-glycolipids and betaine lipids [4,8]. They are connected to the polar radicals of molecules by ester or amide bonds. Membrane VLCFA can be saturated and unsaturated. These VLCFA are essential participants in many biological processes that cannot proceed by means of C_14–18_ aliphatic chains alone [4,9].

There is an obvious connection between the growth, division and differentiation of plant cells and the processes of VLCFA biosynthesis [6]. With a violation of the level of VLCFA in plants that are mutants lacking genes involved in the synthesis of VLCFA, or, conversely, when these genes are overexpressed, phenotypic changes occur, leading to growth retardation and even death of the embryos [3,4,10,11,12]. VLCFA-containing PhL play an important role in the dynamics of endomembranes during cytokinesis and cell differentiation [13]. There are data indicating the possibility of VLCFA playing the role of signaling molecules in resisting various types of stress [7,14,15,16]. It was found that auxin signal transduction mediated by mitogen-activated protein kinase 14 (MAPK14), with the participation of ethylene-dependent transcription factor ERF13, modulates the development of plant lateral roots through the biosynthesis of new VLCFA [17].

A surface barrier to protect terrestrial plants from a range of biotic and abiotic stresses is created by the cuticle, which is a continuous lipophilic layer covering the surfaces of all types of epidermal cells of terrestrial plants. The cuticle is composed of surface cuticular waxes, cutin and suberin [18]. In this case, cuticular waxes can be both epicuticular and intracuticular, which can be embedded inside the cutin layer bordering the primary cell wall and built into it. Cutin does not contain VLCFA and includes only C_16–18_ hydroxy- and epoxy-FA [19].

Cuticular waxes consist mainly of n-alkanes, ketones, secondary alcohols and aldehydes, each of which has a chain length of C_21–35_, as well as fatty alcohols with C_22–34_. Cuticular waxes also include free VLCFA together with C_16–18_ fatty acids and the VLCFA linked by an ester bond with alcohols in the wax esters. In cuticular waxes and the pollen coat, the total length of the VLCFA chain, together with higher alcohols, can be C_38–50_ [20]. According to other data, wax esters are formed mainly from alcohol C_28_ and FA C_16–20_, forming chains with C_42–52_ [21]. The amount of free VLCFA and their esters with alcohols in the structures of cuticular waxes is usually small; thus, the stems of Arabidopsis (*Arabidopsis thaliana* Heynh. (L.) contain 4% C_16–34_ FA, together with wax esters containing the same acids [22]. The aliphatic chains of cuticular waxes are mostly saturated [2]. It is important that the newly formed C_20–38_ VLCFA, together with C_16–18_ FA, serve as substrates for enzymes synthesizing the main aliphatic components of the cuticular waxes, suberin and pollen coats listed above [1,6,23].

Suberin, as part of the cuticle, is found on the inner surfaces of the primary cell walls of various plant parts, including the peridermal tissues of shoots and roots and the root endoderm. It is a polyester of glycerin, phenols and fatty acid derivatives. The aliphatic part of suberin consists of C_16–18_ hydroxy-FA, C_16–26_ fatty α, ω-diacids, C_18–30_ alcohols, a small amount of free VLCFA and wax esters consisting of VLCFA and higher alcohols [18,20]. Sporopollenin is the main constituent of the outer pollen coat. Cytochrome P450, labeled as CYP704 B1, was identified in Arabidopsis, which is necessary to ensure the synthesis of a number of components derived from VLCFA and which are the building materials for sporopollenin. The synthesis of the latter requires the expression of the *CYP703A2* gene, as well as *STERILITY2*, which encodes FA acyl-reductase [24].

Finally, VLCFA can be contained in developing seeds, accounting for up to two-thirds of the total amount of FA. There, they can be in the composition of triacylglycerols (TAG), as in *Brassica napus* seeds, or esters with fatty alcohols, as in *Simmondsia chinensis*, in which the total length of the hydrocarbon chain can reach C_32–64_ [22]. Thus, VLCFA can occur in small amounts in cuticular waxes [21] or suberin [20] in the free form or in the form of their methyl esters. Most often, VLCFA occur in a bound form, forming ester or amide bonds with partners. It is known that the VLCFA synthesized in the endoplasmic reticulum (ER) are included in the three main lipid pools: (1) polar lipids (PL); (2) storage TAGs or waxes and (3) cuticular waxes, suberin and sporopollenin [4,7].

## 2. The Initial Stages of VLCFA Synthesis

The participation of VLCFA in many metabolic processes in plants has aroused interest in the biosynthesis of these compounds [2,6,7,25,26]. Information concerning the synthesis of VLCFA, in contrast to regular-chain FA, is rather poor [9,27,28]. It can be seen that, in a number of plant objects, the elongation process stops at the level of C_18_ FA (according to the gas–liquid chromatography (GLC) analysis of the total FA); in other objects, it continues up to C_20–22_, and in others, it lasts much further (up to C_26–28_). In addition, in most terrestrial plants with a cuticle or suberin, the biosynthesis of VLCFA can continue until the formation of C_34–38_ [7]. The mechanisms of VLCFA synthesis are often extrapolated from the data or speculations that are available in relation to the elongases and desaturases of mammals or bacteria and fungi [29,30]. There is only the initial information about the biosynthesis of VLCFA in plants. For example, it is known that saturated and monounsaturated VLCFA can be formed by elongations of 18:0, 18:1 and 20:1, respectively [2,26,27,31,32]. Labeled VLCFA-CoA and then labeled glycerolipids containing these VLCFA were obtained using the cell homogenates of developing seeds of *Lunaria annua* and labeled malonyl-CoA. It has been shown that in cell homogenates occur the same reactions of VLCFA synthesis, which are typical for animal tissues, and VLCFA 20:0 and unsaturated up to 22:1^∆9^ are formed by elongation and desaturation in the same way as regular FA [33].

The process of C_26–38_ VLCFA elongation is even less studied. Little is known about the proteins involved in the formation of such VLCFA, which are necessary for the further synthesis of waxes [1]. The key condensing enzymes required for the elongation of VLCFA cannot effectively synthesize FA with a length of more than 28 atoms of C. At the same time, C_28–34_ acyl lipids predominate in cuticular waxes and the pollen coat [34], while alkanes C_35_ and C_37_ in *A. thaliana* leaf trichomes are obtained from acyl-CoA C_36–38_ [1]. It is known that VLCFA elongation in the ER is carried out by multiple FA elongase complexes with different substrate specificities, adapted to the synthesis of a number of products intended for different metabolic pathways. Such products can be, in particular, C_28–38_ VLCFA, which are consumed immediately after their formation for the synthesis of n-, iso- and anteisoalkanes; ketones; aldehydes and higher alcohols that make up cuticular waxes, pollen coats and suberin [6,7,34].

Recently, genes encoding enzymes involved in the synthesis of both regular-length FA and VLCFA have been discovered and studied [6]. As a result of sequencing the transcriptome of the epidermis of terrestrial plants, a list of potential candidate genes involved in the synthesis of surface lipids was obtained [35]. The identification and description of the elongation enzymes of both regular FA and VLCFA, as well as the genes encoding these enzymes, became possible as a result of biochemical and genetic studies of model organisms—yeast (*Saccharomyces cerevisiae*) and Arabidopsis. Progress in the study of the biochemistry of plant elongases is modest due to their complicated heterological expression, difficulties with the conversion to a soluble state and purification of these enzymes associated with membranes [36]. The achieved degree of purification of elongases made it possible to obtain some information about their structure, functioning and the effect of the lipid environment of the enzyme in the membrane on its activity [2,36].

**Figure 2:**
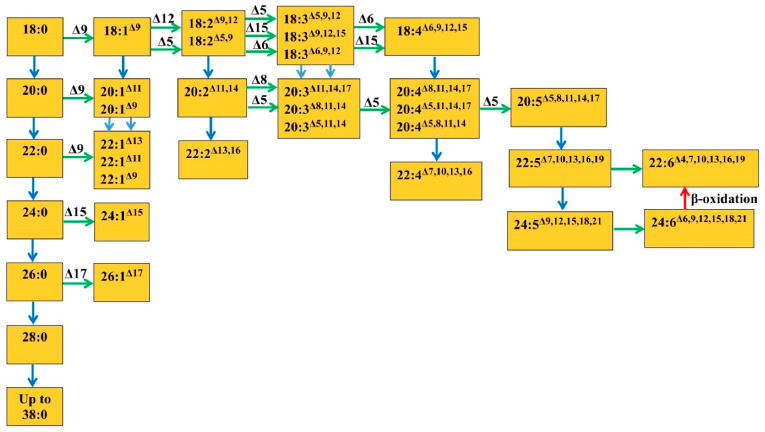
Metabolic pathways of VLCFA synthesis in plant tissues. The presented scheme shows the main pathways for the biosynthesis of saturated and unsaturated VLCFA, starting with their precursors—18:0 and 18:1 fatty acids (FA). It can be seen that, often, the final product is obtained not in one way but in several. Thus, unsaturated VLCFA can be obtained either by desaturation or by elongation of the previous unsaturated products. It is assumed that the processes of desaturation and elongation of VLCFA can alternate. Different final products can be synthesized from the same intermediate compounds. Vertical arrow—C_2_-elongation and horizontal arrow-desaturation.

The presented hypothetical scheme (Figure 2) shows the most studied ways of the biosynthesis of saturated and unsaturated VLCFA, starting with their precursors—18:0 and 18:1—in plant tissues. This includes the VLCFA biosynthesis pathways known for euglenic algae and microalgae. It can be seen from the scheme that, often, the final product is obtained not in one way but in several. Thus, an unsaturated acid can be obtained either by desaturation or by elongation of the previous unsaturated products. It is assumed that the processes of desaturation and elongation of VLCFA may alternate [37]. Saturated and unsaturated VLCFA undergo elongation, probably with the help of different enzymes, and the differences in their mechanisms of action have not been studied enough yet. Participation in the process of the transformation of various enzymes, their different isoforms or different precursors can lead to different ways of synthesizing the same product, or vice versa, different final products can be synthesized from the same intermediate compounds [32].

The adaptation of FA metabolism to stress conditions (in particular, cadmium stress) may require such regulatory processes as lipolytic or peroxidative activity. The catabolic process of β-oxidation of VLCFA can take place up to palmitic acid. Moreover, this process can be reversed not only to the elongation of saturated FA but also to the desaturation and elongation of unsaturated FA. The peroxisomal β-oxidation of fatty acids takes place during the breakdown of storage oil in germinating oilseeds. Long-chain acyl-CoA synthetases (LACS) are involved in this process, activating the formed free FA [23]. Two genes (*AtLACS6* and *AtLACS7*) from *A. thaliana* encoding LACS proteins capable of initiating β-oxidation in plant peroxisomes have been identified [38].

There are metabolic difficulties in the implementation of VLCFA biosynthesis associated with the fact that the elongation of the FA chain occurs in one place (in the ER), and desaturation of up to three double bonds occurs in another (in plastids) [28]. The lipid traffic from ER to plastids is mediated by LACS9 and LACS4 [39]. The TGD4 protein of Arabidopsis, which is present in the outer membrane of chloroplasts, transfers phosphatidic acid from the ER either only up to this membrane or through it into the chloroplast [40].

The biosynthesis of FA begins with the formation of acetyl-CoA in plastids, which possesses the ATP necessary for it [30,41]. The first enzyme in FA biosynthesis is acetyl-CoA carboxylase (ACC), the rate of which determines the rate of lipid synthesis in general [26]. This enzyme catalyzes the ATP-dependent carboxylation of acetyl-CoA with the formation of malonyl-CoA, which is further used both in plastids for the synthesis of regular-chain FA and in ER in various biosynthetic pathways, including the elongation of VLCFA to C_24–28_. It has been shown that the continuous synthesis of malonyl-CoA occurs in yeast with the participation of the ACC enzyme for the formation of C_26_ VLCFA, which, in turn, is necessary for the normal functioning of the nuclear membrane [42].

The ACC complex in plants can exist in two structurally distinct forms: a multifunctional homodimeric form with subunits > 200 kDa and a dissociable, multi-subunit, heteromeric form made up of four proteins. The multi-subunit form is present in plastids of dicots and non-Poacae monocots, whereas the multifunctional form is thought to be a cytosolic form, with the exception of Poacae, which possess a multifunctional form in their plastids [41]. *A. thaliana* has two nuclear genes (*ACC1*, also known as *GURKE*, and *ACC2*) that encode two ACC isoforms. Two mutants with damage to the *ACC1* gene were isolated and characterized. Both *acc1–1* and *acc1-2* mutations are recessive and impair cotyledon formation. At the same time, all lipid fractions of the abnormal embryo lack VLCFA and are enriched with C_18:1_. Thus, the functioning of ACC is associated with the elongation of FA to the VLCFA [41].

It is known that the synthesis of regular-length FA in plastids is carried out using an elongase system having three forms responsible for the alternate formation of 4:0-CoA, a series from 6:0-CoA to 16:0-CoA inclusively and 18:0-CoA. These forms are β-ketoacyl-ACP synthases III, I and II, respectively (KAS III, I and II), where ACP is an acyl carrier protein [26,28,43,44,45,46,47]. Each of these FA elongation systems functioning in plastids involves a cycle of four reactions that are catalyzed by the following enzymes: β-ketoacyl-CoA-synthase (KCS), β-ketoacyl-CoA-reductase (KCR), β-hydroxyacyl-CoA-dehydratase (HCD) and *trans*-2,3-enoyl-CoA-reductase (ECR). It is assumed that all four enzymes of the elongase system involved in the biosynthesis of regular FA exist in the form of an aggregate located around an acyl carrier protein (ACP), which transfers the synthesis products within the elongase system from one enzyme to another [6,25].

Until recently, it has not been definitively established which of these enzymes is responsible for the substrate specificity and limits the reaction rate. The biochemical characteristics of membrane-bound elongation enzymes have not been sufficiently studied yet; however, there is evidence that the rate of the process depends on KCS synthase, which is often called a condensing enzyme. This enzyme performs the first stage of FA elongation in plastids, which consists of the condensation reaction of acyl-CoA and malonyl-ACP [48]. It is assumed that KCS performs the same functions in the synthesis of VLCFA in the ER [49].

## 3. VLCFA Chain Elongation in ER

It is known that, at the final stage of the biosynthesis of regular FA, 16:0, 18:0 and 16:1 and 18:1 FA formed from acyl-ACP after hydrolysis of the latter by acyl-ACP thioesterases are exported from plastids and converted under the action of LACS into acyl-CoA and are transferred to the ER membranes [50,51,52,53,54]. Enzymes of the LACS class are involved in several metabolic pathways starting from FA, including the biosynthesis of PL, TAG, jasmonate and β-oxidation of FA [52,53].

The family, which includes nine genes, encodes LACS in Arabidopsis. Among them, the LACS9 isoform localized on the outer plastid membrane [6] and highly expressed in developing seeds and young leaf rosettes was identified [52]. In addition to LACS9, synthetases LACS1, LACS2, LACS4 and LACS8 were found in Arabidopsis seeds, which were associated with plastids or the ER [52,53,54]. An analysis of the mutant lines showed that the functions of LACS9 synthetase in relation to the transfer of lipids from the ER to plastids largely overlap with those of LACS4 [39]. LACS1 and LACS2 in vitro provided the binding of C_20–30_ VLCFA to CoA [6,23]. Double mutants for the *LACS1/CER8* and *LACS2* genes showed the effects associated with cuticular wax deficiency and organ fusion, so it is clear that LACS1 and LACS2 are jointly involved in the preparation for cuticular wax biosynthesis [6]. In addition to the fact that the LACS1 isoform localized in the ER is the main one on which the formation of cuticular lipids depends, it functionally overlaps with LACS9 during TAG biosynthesis [55].

C_16_- and C_18_-CoA participate in the synthesis of polar lipids and elongate to VLCFA in the ER [26]. What the VLCFA biosynthetic machine consists of and how it provides structural diversity is not completely understood [56]. When discussing the synthesis of VLCFA in the ER, the works of a number of authors listed the same processes that occur during the synthesis of regular FA in plastids. Therefore, it is important to establish whether there are any differences in the structure and functions of the elongase ER system compared to that localized in plastids. There is indirect evidence that higher plants have a variety of elongation systems in the ER specific in relation to the length of the chain. Probably, there are different elongation systems responsible for the elongation of either saturated or unsaturated VLCFA [2,25].

The mechanism of the formation of saturated FA with an even number of C atoms (C_20–24_) in higher plants is currently described in sufficient detail (Figure 2) [2,25,33,57]. It consists of the standard C_2_-elongation of 18:0 and 20:0 acyl-CoA derivatives previously synthesized in plastids, using an elongase system located in the ER, in the presence of malonyl-CoA as a C_2_ donor. Batsale et al. [7] showed that the synthesis of saturated VLCFA in plants goes not only up to C_26_ but up to C_38_. It is considered that saturated VLCFA with an odd number of C atoms—21:0, 23:0 and 25:0—found in the cell membranes of the onion epidermis are products of the α-oxidation of saturated “even” VLCFA [2]. It is also assumed that “odd” plant FA, similar to “even” ones, can arise in the C2-elongation reaction if propionyl-CoA is used instead of acyl-CoA as the primer of this reaction [58]. One cannot rule out the possibility of the formation of “odd” VLCFA by β-oxidation in peroxisomes [25,38]. Experimentally, the formation of 23:0 and 25:0 out of 21:0 was found only in the yeast *Candida utilis* [58].

The study of VLCFA biosynthesis in higher plants in vivo by the insertion of [1-^14^C]-acetate in the tissues of cereal seedlings showed that VLCFA formation occurred due to elongation rather than de novo synthesis. An analysis of the label distribution in VLCFA synthesized from [1-^14^C]-acetate in the epidermis of leeks suggests the involvement of multiple elongation systems [2] and, first of all, in the ER [25]. After treatment with various inhibitors, the order of the in vitro elongation of VLCFA in potatoes indicates that this process is controlled by three separate elongases. Subsequent genetic studies have confirmed the presence of various elongation systems in higher plants [2,6]. Further transformations of polyunsaturated VLCFA into ER have been carried out using multiple complexes of heterotetrametric elongases that catalyze the gradual addition of 2C to the acyl-CoA substrate. It is accepted that VLCFA are synthesized by the serial elongation of precursors using the ER elongase complex [4,6], similar to the same complex existing in plastids, in the course of a repeated cycle of the four subsequent enzymatic reactions (see above). It is recognized that the mechanism of synthesis of VLCFA is cyclic; the presence of a mutationally regulated circular mechanism that generates an assortment of cellular VLCFA has been shown. Thus, proteoliposomes assembled from purified membrane components—the elongase protein (Elop), dehydratase and two reductases—catalyzed the repeated cycles of the addition of two C atoms, which extended the regular FA and turned them into VLCFA whose length was dictated by the presence of a specific Elop homolog [9].

Currently, our knowledge of the specific role of various elongation enzymes is insufficient. In general, the stoichiometry and quaternary structures of the VLCFA elongation complex are still unclear, and phylogenetic and biochemical questions for each component of this complex remain unresolved [6]. It remains to be elucidated how the process of VLCFA elongation can be regulated at the posttranscriptional level [6]. Despite the fact that many genes involved in the regulation of VLCFA synthesis have been studied using direct and reverse genetics methods, it is obvious that there are other genes that have yet to be discovered.

## 4. Functioning of Enzymes of the VLCFA Elongase System in the ER

**Figure 3:**
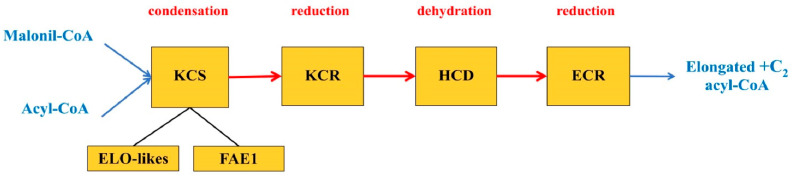
Enzymes of the VLCFA elongase complex. The elongation of VLCFA in the endoplasmic reticulum (ER) is carried out by an elongase complex that consists of four enzymes: β-ketoacyl-CoA-synthase (KCS), β-ketoacyl-CoA-reductase (KCR), β-hydroxyacyl-CoA-dehydratase (HCD) and enoyl-CoA-reductase (ECR), which catalyze four consecutive reactions: a condensation, a reduction, a dehydration and a final reduction, respectively. All these reactions give an acyl-CoA that is two carbon atoms longer than the initial acyl-CoA. ELONGATION DEFECTIVE-likes (ELO-likes) and FATTY ACID ELONGATION 1 (FAE1) are two families of condensing enzymes.

The VLCFA elongation complex consists of four enzymes localized in the ER (Figure 3). KCS is a key component of the elongase system in plants and regulates the substrate and tissue specificity for VLCFA by the chain length and even by the degree of unsaturation [10,25,49]. Identification and characterization of the four enzymes of the elongase complex in *A. thaliana* showed that KCS participates in the biosynthesis of VLCFA and determines the amount of the product synthesized by the whole complex, while the other three enzymes are universal and perform their standard functions in the biosynthesis of these compounds [6].

The study of the structural and functional features of elongase systems of plants allowed us to divide their condensing enzymes (KCS) into two families: ELONGATION DEFECTIVE-likes (ELO-likes) and FATTY ACID ELONGATION 1 (FAE1) [6,11,50]. If the first ones are present in all living organisms and participate in the formation of a long chain of FA, then the second ones are found only in plants and provide the formation of waxes and storage lipids of seeds. Presumably, FAE1 enzymes are structurally unrelated to ELO-like enzymes [25]. Thus, the genes for both of these enzymes are found in all terrestrial plants [11,50]. The list of known genes involved in the synthesis of VLCFA in Arabidopsis is given in a number of works [4,6,14,19]. An analysis of the subcellular localization of the KCS family proteins in tobacco (*Nicotiana tabacum* L.) cells showed that all of them are located in the ER; therefore, VLCFA synthesis occurs in this cell region [59]. Angiosperms have 26 genomes and two transcriptomes that encode KCS [50]. For example, in Arabidopsis, the ELO-likes family includes four genes, while the FAE1 family includes 21 genes [4], and 58 FAE1 genes have been identified in the *Brassica napus* genome, while only 14 ELO-like genes have been found [51].

ELO-like plant proteins are homologs of yeast ELO proteins, which are studied quite well. ELO enzymes have different substrate specificities, which determine the chain length and the degree of unsaturation of the VLCFA they synthesize. The heterologous expression of Elop homologs in yeast has shown that these proteins determine the length of VLCFA produced by cells [9]. It is known that KCS proteins that are encoded by the multigene *ELO* family perform VLCFA elongation in different ways. In yeast, Elop2 is responsible for the elongation of VLCFA to C_24_, and Elop3 is required for the conversion from C_24_ to C_26_ [4].

Among ELO enzymes with different substrate specificities, the position of the lysine residue in the molecule varies. The mutational analysis showed that the active site of Elop is turned into the cytosol, and the length of the VLCFA is determined by the lysine residue near the luminal end of the transmembrane helix of Elop. By stepwise moving the lysine residue along one surface of the helix towards the cytosol, new KCS synthases were constructed that produce correspondingly shorter VLCFA. It is assumed that the charged lysine residue limits the chain length of the hydrophobic FA substrate, which can fit inside the substrate-binding pocket. Site-directed mutagenesis was used to move the lysine in Elo3p deeper into the pocket that produces C_26_-VLCFA. This increased the number of synthesized VLCFA molecules from C_26_ to C_30_, confirming the dependence of the chain length on the distance between the active site and the lysine residue [6,9]. It is possible that Elops are a family of condensing enzymes that precisely determine the length of VLCFA, limiting the number of elongation cycles after products of a certain length are obtained [6].

The information about the second family of condensing enzymes—FAE1 (from KCS1 to KCS21), identified in Arabidopsis—has been given in some papers [4,6,14]. The Arabidopsis *FAE1/KCS18* gene encodes a seed-specific condensing enzyme responsible for the elongation of C_20–22_ VLCFA into a TAG. The *fae1* mutation led to a reduction in the level of VLCFA in seeds from the initial 28% to less than 1% and affected only the composition of the FA of developing seeds without affecting the composition of the FA in the vegetative part of the plant or flowers [27]. Expression of the *FAE1/KCS18* condensing enzyme gene in the leaves of transgenic *35S-FAE1* Arabidopsis plants resulted in a significant accumulation of C_20_ and C_22_ VLCFA. Thus, it was shown that the expression of only one *FAE1/KCS18* gene was sufficient to ensure the synthesis of VLCFA in those tissues where they are usually not found [27]. *FAE1/KCS18* homologs were also found in Brassicaceae and Simmondsiaceae, and the participation of this gene and the corresponding elongase in the synthesis of erucic acid was shown (22:1) [25].

In addition to the *FAE1/KCS18* gene, the *CER6/KCS6* gene is described in detail in Arabidopsis, which controls the elongation of C_≥24_ VLCFA specific for epidermal cells [14,27]. The functions of the other condensing enzymes in VLCFA elongation are less studied [4,10]. The KCS genes: *CER6/KCS6*, *KCS1* and *FIDDLEHEAD* (*FDH/KCS10*) are involved in the synthesis of VLCFA as precursors of the shoot wax components. Two additional KCS genes, *KCS20* and *KCS2/DAISY*, are required for the elongation of C_20_ to C_22_ VLCFA during cuticular wax and suberin biosynthesis in roots [4,60]. The Arabidopsis mutant, *daisy*, had disturbances during root growth, and the content of C_22–24_ VLCFA in suberin decreased while the content of C_16–20_ FA increased [61]. Studies of the *FAE1* genes family, such as *FDH/KCS10* and *HIC/KCS13*, have shown that they play an important role in morphogenesis and adaptation to the environment in Arabidopsis, but a clear relationship between the defective synthesis of VLCFA and the phenotypes of the *FDH* and *HIC* mutants has not been established [10,14].

The *KCS9* gene showed the highest expression in the cells of the Arabidopsis stem epidermis. The *kcs9* knockout mutants showed a significant decrease in C_24_ VLCFA and, conversely, accumulation of C_20–22_ VLCFA in the membrane and surface lipids. Thus, KCS9 is involved in the elongation of C_22_ VLCFA to C_24_, which are important precursors for the biosynthesis of cuticular waxes, aliphatic suberins and membrane lipids, including PhL and sphingolipids [5,62].

Thirty *AhKCS* genes have been identified in the peanut genome (*Arachis hypogaea* L.). Nine *AhKCS* genes that are highly expressed in developing seeds have been cloned; each of them catalyzes the elongation of VLCFA with different substrate specificities. *AhKCS1* and *AhKCS28* had the highest expression levels; probably, these genes are involved in the regulation of the content of VLCFA in peanut seeds. The overexpression of these genes in Arabidopsis greatly increased the content of VLCFA, especially saturated ones, in seeds [63]. The participation of the *Wax Cristal-Sparse Leaf1* (*WSL1*) gene in the VLCFA elongation to C_20–24_ and the biosynthesis of cuticular waxes in rice leaves has been shown [64]. The *cer-zh* barley (*Hordeum vulgare*) mutants have less or no epicuticular wax crystals on the leaves. The heterologous expression in yeast showed that CER-ZH/*Hv*KCS1 has a substrate specificity for C_16–20_, especially unsaturated ones, which plays an important role in the elongation of acyl chains for wax biosynthesis [65]. The *emr1/Hvkcs6* barley mutants have significantly less aliphatic wax components, with a chain length of more than C_24_ [66].

Elongases operating with saturated FA are designated as Δ0-ELO and with a polyunsaturated substrate as Δ6-ELO when the substrate has a double bond in the corresponding position. Elongase 16:0 has been shown to affect the levels of 22:5 VLCFA plastid monogalactosyldiacylglycerols (MGDG) in Nannochloropsis. If VLCFA are formed in the ER, therefore, the formation of MGDG depends on the entry of 22:5 from the ER into the plastid as a result of some unknown processes. Seven elongases and five desaturases have been identified, possibly involved in the formation of 22:5 in *N. gaditana*. The Δ0-ELO1 elongase isoform, capable of elongating 16:0, turned out to be actively expressed. It is suggested that, in Nannochloropsis, the part of 22:5 used for the formation of MGDG is synthesized as the result of a process initiated at the 16:0 elongation stage using Δ0-ELO1, which thereby acts as a proxy enzyme for the synthesis of galactolipids [67].

The second component of the elongase complex is β-ketoacyl-CoA reductase (KCR), which catalyzes the first reduction during the elongation of VLCFA. A study of the Arabidopsis genome revealed two sequences homologous to *YBR159w* encoding KCR in yeast (*Saccharomyces cerevisiae*). Both the *AtKCR1* and *AtKCR2* genes were transcribed in many plant organs, but only the *KCR1* transcript was found in the roots. It turned out that only *KCR1* is a functional form of KCR involved in VLCFA elongation in the ER. The suppressed activity of *KCR1* leads to a decrease in the content of cuticular waxes and affects the composition of VLCFA sphingolipids, seed TAG and root glycerolipids, showing that KCR participates in elongation reactions, supplying these classes of lipids with VLCFA [68]. In maize, KCR is encoded by two genes, *GL8A* and *GL8B*. It turned out that the double mutant *gl8agl8b* is fatal for the embryo, which shows the crucial role of VLCFA for the full embryonic development of plants [69].

β-Hydroxyacyl-CoA dehydratase (HCD) is the third enzyme of the elongase complex and is a necessary enzyme for the synthesis of VLCFA. HCD is encoded by the *PASTICCINO2* (*PAS2*) gene in Arabidopsis, which is a homolog of the previously identified *YL097/w/PHS1* yeast β-hydroxyacyl-CoA dehydratase (PHS1) gene [14]. The *PHS1* gene is essential for the vital activity of yeast cells during cell division. The *PAS2* gene is very important for the normal development of the embryo in Arabidopsis, since the complete loss of its activity was fatal for the embryo [43]. The partial loss of *PAS2* function led to a general reduction in the number of VLCFA in the composition of TAG, complex sphingolipids and cuticular waxes, and at the same time, there was an accumulation of long-chain bases and precursors of sphingolipids [4,11]. The *pas2-1* mutant of Arabidopsis was characterized by a general decrease in the VLCFA pools in the storage TAGs of seeds, cuticular waxes and complex sphingolipids. Damage to the elongation cycle led to the accumulation of 3-hydroxyacyl-CoA intermediates, indicating the premature termination of FA elongation and confirming the role of *PAS2* in this process [41]. Previously, the KCS enzyme was considered the only limiting link in the elongation of VLCFA, therefore, it is interesting that the overexpression of dehydratase activity in Arabidopsis leads to an increase in the VLCFA content mainly in cuticular waxes. This suggests that the elongase complex contains several limiting enzymatic steps [2,4].

Finally, the Arabidopsis ECERIFERUM10 (CER10) protein was identified as a candidate for the fourth enzyme of the elongase complex based on the similarity of the *CER10* gene to the yeast *TSC13* gene encoding *trans*-2,3-enoyl-CoA reductase (ECR). Arabidopsis CER10 is localized in the ER. Using Arabidopsis *cer10-1* and *cer10-2* mutants, it was shown that the *CER10* gene product is involved in the VLCFA elongation. Damage to this gene has a serious impact on the growth and morphogenesis of the shoot, but the loss of CER10 is not lethal for plants [10]. Since the *CER 10* gene is not essential in contrast to *KCR1* or *PAS2*, this indicates the existence of other functionally equivalent ECR isoforms in Arabidopsis [4].

Thus, at present, very little is known about the genes encoding individual enzymes that are involved in several stages of VLCFA biosynthesis. Some authors believe that it is almost impossible to establish a correspondence between genes and biochemical processes. The synthesis of the polypeptide chains of one enzyme can be encoded by different genes located on different chromosomes or even in different parental genomes. Taking into account the complexity of the regulation of the work of genes in various tissues, it can be assumed that the normal functions of each block of synthesis can be determined not by individual genes but by gene networks consisting of many structural and regulatory genes [32].

## 5. Double Bond Formation in VLCFA

VLCFA, having very long acyl chains, can include from one to eight double bonds. In the case of VLCFA synthesis from regular unsaturated fatty acids, the first two double bonds in VLCFA are usually transferred together with the precursors. Thus, in the seeds of the Cruciferae family, TAGs contain a significant amount of mono- and diunsaturated VLCFA. It is assumed that one of the pathways for the biosynthesis of these fatty acids consists of a sequential stepwise C_2_-elongation of 18:1^Δ9^ and 18:2^Δ9,12^, which leads to the formation of VLCFA 20:1^Δ11^, 22:1^Δ13^ and 20:2^Δ11,14^ present in rape seeds, as well as 22:2^Δ13,16^. This pathway may have a further continuation [33] (Figure 2). The further desaturation of acyl residues included in PhL may occur in ER membranes. In this case, the resulting 18:2 and 18:3 FA residues that replace the C_2_- position of glycerol can be exchanged for an 18:1 acyl radical, followed by the introduction of new double bonds [26].

The formation of double bonds in FA molecules is catalyzed by specific acetyl-CoA oxygenases or desaturases. The first double bond is inserted into the newly formed FA by stearoyl-ACP desaturase (Δ9-desaturase), which is a soluble protein localized in plastids [26]. In addition to this type of soluble acyl-ACP Δ9-desaturase, plants and algae also have a second type, acyl-lipid Δ9-desaturase, which probably acts both in thylakoids and in the ER [70]. In addition to Δ9-stearoyl- or palmitoyl-ACP desaturases, different plants also contain other soluble acyl-ACP desaturases: Δ4-, Δ5- and Δ6-palmitoyl-ACP or Δ9-myristoyl-ACP desaturases, which are localized in the stroma of chloroplasts. Some plants possess additional desaturases, which, being structurally similar to ∆9-desaturase, introduce an ethylene bond into a different position of the acyl chains and may have a different substrate specificity. Thus, as a result of the action of plastid Δ4-desaturase, petroselinic acid (∆6-18:1) is formed [71].

The second double bond can be formed in the FA molecules of plants both in plastids and outside them in the ER. Two acyl-lipid Δ12-desaturases are involved in the formation of the second double bond. If Fad2 desaturase acts in the ER, using, in particular, phosphatidylcholine (PC), then Fad6, associated with glycolipids, works in the thylakoid membranes of chloroplasts. These enzymes are also called ω6-desaturases, and they form linoleic acid (18:2^Δ9,12^) [72]. Two Δ12 desaturase genes, *fad2* and *fad6*, encoding microsomal and chloroplast Δ12 desaturases, respectively, have been identified in the *A. thaliana* genome [2]. In addition, Fad2 is localized in cytoplasmic membranes, as well as in the ER [73]. Fad2 desaturase in Arabidopsis is present in the singular, whereas in cotton or soy, it is represented by several (up to four) isomers. Different isoforms of the same enzyme can participate in synthesis at different stages. Thus, two forms of the *fad* gene encoding the formation of a double bond at the ninth atom of C were found in soy: *fad2-1* and *fad2-2*. The first is intensively expressed only in ripening seeds and the second in vegetative tissues and seeds [32].

Thus, in addition to soluble acyl-ACP desaturases, there are also membrane acyl-lipid desaturases in plants (in the ER): acyl-lipid I- and acyl-lipid II-desaturases [73]. With the help of these desaturases, which are integral proteins of membranes of the ER and react only with FA radicals included in the composition of membrane lipids, additional double bonds are introduced [12]. Desaturases of the ER form double bonds in FA esterified at any of the sn-positions of phosphatidic acid, PC or another polar lipid [73]. The subcellular localization of two integral membrane-bound FA desaturases, Fad2 and Fad3, was determined by an immunofluorescence microscopic analysis of tobacco suspension cells. N-terminal zones of both Fad2 and Fad3 were shown to be exposed on the cytosolic side of the ER membranes [74].

It is known that the processes of the desaturation and elongation of FA can often alternate [37]. In this case, desaturation is catalyzed by anterior or frontal desaturases, which introduce double bonds between the already existing double bond and the carboxyl (frontal) end of unsaturated FA. Therefore, the Δ6-acyl-lipid frontal desaturase turned out to be necessary for the formation of γ-linolenic acid (18:3^Δ6,9,12^) [75]. These desaturases have been identified and characterized in a wide range of eukaryotic species, including plants and animals. The *PiDesD6* and *PiDesD5* genes for frontal acyl-lipid desaturases Δ6 and Δ5 were isolated from the microalgae *Parietochloris incise*, and the nucleotide sequence was determined [76]. Unlike frontal bacterial desaturases, eukaryotes are structurally characterized by the presence of an N-terminal cytochrome b_5_-like domain connected to the main desaturase domain [37].

The enzyme that forms the third double bond is acyl-lipid Δ15-linoleic acid desaturase (Fad3), which is involved in the synthesis of α-linolenic acid (18:3^Δ9,12,15^). This enzyme is often referred to as ω3-desaturase. The T-DNA gene for microsomal ω-3 FA desaturase was isolated from Arabidopsis using the cDNA-labeling method. It was shown that the enzyme encoded by this gene limits the formation of 18:3 in the seeds of transgenic Arabidopsis [77]. From the family of Δ15-desaturases in Arabidopsis, in addition to Fad3, which functions on phosphatidylcholine in the ER, there are also Fad7 and Fad8 acting on glycolipids of chloroplasts [2]. It can be noted that Δ4- and Δ8-acyl-CoA desaturases are present in *Euglena*, while Δ6 is found in borage (*Borago oficinalis*) [73,75]. The frontal desaturases Δ6 and Δ4 were found in the photoautotrophic green microalgae *Ostreococcus* RCC809 and diatom algae *Fragilariopsis cylindrus*. It has been shown that Δ4-desaturase participates in the formation of 22:5 [78]. Acyl-lipid Δ7-desaturase was also found in plants. The Arabidopsis gene *AtADS3/FAD5*, encoding plastid palmitoyl-MGDG Δ7-desaturase, is also known [79]. In fungi, Δ17-desaturase was found, which acts on 20:3^Δ8,11,14^ and 20:4^Δ5,8,11,14^. In addition, possible ways of the biosynthesis of 28:7 and 28:8 VLCFA, as well as 27:6, 27:7 and 27:8, in dinoflagellates are known [73].

The study of the family of nine Arabidopsis acyl-CoA desaturase-like (*ADS*) genes encoding proteins similar to FA desaturase showed that seven of them exhibit different specificities, catalyzing the introduction of double bonds relative to the methyl end of the molecule at n-6, n-7 and n-9. Using forward and reverse genetic methods, it was shown that one of these genes, *ADS2*, is involved in the synthesis of the C_24:1_ (n-9) and C_26:1_ (n-9) components of seed lipids, sphingolipids and membrane PhL. The overexpression of *ADS2* led to a significant increase in the relative contents of the lipid classes containing C_24:1_ and a sharp increase in the number of monounsaturated VLCFA in the acyl-CoA pool. It is assumed that the monounsaturated C_24–26_ VLCFA of Arabidopsis are the result mainly of the desaturation of saturated VLCFA rather than the elongation of shorter monounsaturated acids [79]. However, it would be premature to exclude the possibility of the synthesis of these VLCFA by elongation (Figure 2).

Δ6- and Δ5-desaturases are involved in the biosynthesis of VLCFA in mammalian tissues that use exogenous FA (18:2 and 18:3), and the synthesis reaches 24:6, followed by β-oxidation to docosahexaenoic acid 22:6^Δ4.7,10,13,16,19^. This is the so-called “classical” way of VLCFA synthesis [25]. Since a number of some microalgae produce eicosapentaenoic fatty acid (20:5^Δ5,8,11,14,17^) and a number of others, such as docosahexaenoic, it was assumed that the same standard pathway of VLCFA biosynthesis exists in these organisms as in mammalian tissues. In microalgae the biosynthesis of 22:6^Δ4,7,10,13,16,19^ can occur in two ways, as in mammals. The first consists of elongating 20:5^Δ5,8,11,14,17^ to 22:5^Δ7,10,13,16,19^, which is then desaturated by the Δ4-desaturase to 22:6^Δ4,7,10,13,16,19^ [78,80]. The second begins with two successive cycles of the elongation of 20:5 (see above) and obtaining 24:5^Δ9,12,15,18,21^, followed by desaturation at the Δ6 position and the formation of 24:6^Δ6,9,12,15,18,21^ and, finally, shortening of the chain by β-oxidation in peroxisomes to 22:6^Δ4,7,10,13,16,19^ [25,75].

It is shown that, in plant tissues, the order of formation of C_20_ tri- and tetraunsaturated FA, as well as γ-linolenic acid (18:3^Δ6,9,12^), does not differ from that in microalgae. With the help of Δ6-desaturase, stearidonic acid (18:4^Δ6,9,12,15^) is formed from α-linolenic acid (18:3^Δ9,12,15^). Then, 18:3 and 18:4 acids can be elongated by two C atoms to dihomo-γ-linolenic (20:3^Δ8,11,14^) and eicosatetraenoic (20:4^Δ8,11,14,17^) FA, respectively, and then desaturated with Δ5-desaturase to arachidonic (20:4^Δ5,8,11,14^) and eicosapentaenoic (20:5^Δ5,8,11,14,17^) acids [25,76]. When studying the biosynthesis of 20:5 VLCFA in the microalgae *Porphyridium cruentum* [81], two synthesis pathways were proposed, which involved either Δ5 and Δ17 desaturases or only Δ5 desaturases: (1) 20:3^Δ8,11,14^ → 20:4^Δ5,8,11,14^ → 20:5^Δ5,8,11,14,17^ and (2) 20:4^Δ8,11,14,17^ → 20:5^Δ5,8,11,14,17^.

Tarchevsky and Grechkin [82] reported the same synthesis pathway in higher plants, where 20:4^Δ5,8,11,14^ is synthesized with the help of Δ5 desaturase. In addition, the possibility of the formation of 20:4^Δ5,11,14,17^ from 20:3^Δ11,14,17^ and 20:5^Δ5,8,11,14,17^ from 20:4^Δ8,11,14,17^ in plants was shown (Figure 2). The same pathways for the formation of fatty acids 20:4^Δ5,8,11,14^ and 20:5^Δ5,8,11,14,17^ with the help of Δ5 desaturase in transgenic plants were given in Abbadi et al. [83]. This assumes the synthesis of 20:5 from 20:4 using Δ17 desaturase [83]. In the gymnosperms was found 20:2^Δ5,11^ that formed, most likely, from 20:1^Δ11^ [84]. 20:3^Δ7,11,14^ acid, found in pine (*Pinus silvestris* L.) and synthesized by Δ7 desaturase from 20:2^Δ11,14^ [85], as well as 22:4^Δ7,13,16,19^, is found in lichens (*Parmelia*) and presumably originates from 20:4^Δ5,11,14,17^ [86].

A number of authors have highlighted two ways of the synthesis of VLCFA: the ω6-pathway, from 18:3^Δ6,9,12^ to 20:3^Δ8,11,14^ and 20:4^Δ5,8,11,14^, and the ω3-pathway, from 18:4^Δ6,9,12,15^ to 20:4^Δ8,11,14,17^ and 20:5^Δ5,8,11,14,17^ VLCFA [25,83]. The initial FA for the same two pathways can be 18:2^Δ9,12^ and 18:3^Δ9,12,15^ [87].

The biosynthesis of arachidonic and eicosapentaenoic FA in higher plants was studied using genes encoding enzymes involved in the biosynthetic pathways of ω3/6 Δ8-desaturation for the formation of C_20_ polyunsaturated VLCFA. Arabidopsis was sequentially transformed by genes encoding Δ9-specific elongase activity from *Isochrisis galbana*, Δ8-desaturase from *Euglena gracilis* and Δ5-desaturase from *Mortierella alpina*. An important role in the successful finding of the biosynthetic pathways of polyunsaturated VLCFA is played by the activity of C_18_-Δ9 elongase of *I. galbana*, which can bypass the rate-limiting stages present in the usual pathways of Δ6-desaturase/elongase [87].

The seed-specific expression of the Δ6, Δ5 desaturase and GLELO elongase genes from the filamentous fungus *Mortierella alpina* was performed to increase the synthesis of arachidonic acid in soybean (*Glycine max* (L.) Merr.) seeds. As a result, several FA, such as γ-linolenic, eicosa-8,11-diene, dihomo-γ-linolenic and arachidonic, accumulate in significant amounts [88].

In plants and cyanobacteria, ω3-desaturases catalyze the formation of n-3 VLCFA such as 20:5^Δ5,8,11,14,17^ (eicosapentaenoic) from n-6 VLCFA serving as precursors. The *sdd17* gene encoding a new ω3 desaturase was isolated from the *Saprolegnia diclina* fungus rich in 20:5^Δ5,8,11,14,17^ and characterized. The expression of the resulting gene in yeast (*Saccharomyces cerevisiae*) in the presence of various FA substrates showed that the recombinant protein can desaturate exclusively C_20_ n-6 FA substrates with a clear preference for 20:4^Δ5,8,11,14^ (arachidonic acid), converting it into 20:5^Δ5,8,11,14,17^. The desaturase encoded by *sdd17* also functioned in transgenic somatic soy embryos, resulting in the formation of 20:5^Δ5,8,11,14,17^ from exogenously supplied arachidonic acid, thus demonstrating its potential for use in the synthesis of 20:5^Δ5,8,11,14,17^ in transgenic oilseeds [89].

Euglenic algae (for example, *Euglena gracilis*) have an alternative pathway of VLCFA biosynthesis. It is usually present in organisms that do not show Δ6-desaturase activity. It consists of the elongation of linoleic and α-linolenic acids to 20:2^Δ11,14^ and 20:3^Δ11,14,17^. Further, these VLCFA are transformed into 20:3^Δ8,11,14^ and 20:4^Δ8,11,14,17^ using Δ8-desaturase. The latter are the intermediates of the usual 22:6 biosynthesis pathway. Initially, they undergo further Δ5-desaturation to obtain 20:4^Δ5,8,11,14^ and 20:5^Δ5,8,11,14,17^ acids, elongation to 22:5^Δ7,10,13,16,19^ and Δ4-desaturation to 22:6. This so-called “Δ8-desaturase pathway” has also been previously found in mammals [25]. The *Euglena* Δ8-desaturase gene has been cloned. The Δ8-desaturase from this alga is structurally similar to other desaturases associated with cytochrome b_5_ (Δ6, Δ5 or Δ4) and involved in the synthesis of VLCFA [90].

The relative content of polyunsaturated FA in the eukaryotic lipids of PC and phosphatidylethanolamine (PE) found in the leaves of transgenic 35S-FAE1 Arabidopsis plants is not high, so it can be assumed that microsomal desaturases have low activity with respect to VLCFA. In contrast, unsaturated VLCFA, which are found in chloroplast lipids such as MGDG and digalactosyldiacylglycerols (DGDG), are mostly polyunsaturated, indicating a wide specificity of desaturases with respect to the acyl chain length. Thus, polyunsaturated VLCFA in the leaves of transgenic Arabidopsis arise mainly by the elongation of 18:1 to monounsaturated VLCFA, which are then imported into chloroplasts, where they are further desaturated [3,39].

The resulting transgenic tobacco and flax plants accumulate up to 5% of arachidonic and eicosapentaenoic acids, which are important for humans. It is shown that their further accumulation is difficult, since after Δ6-desaturation, FA are immediately sent to the TAG and are guaranteed to bypass the acyl-CoA pool; as a result of which, the synthesis of elongated C_20_ VLCFA is limited, and the alternating sequence of actions of acyl-lipid desaturases and elongases, the object of which is the mentioned pool, is disrupted [83].

It was found that, during the biosynthesis of alkanes in the ER of leek epidermal cells, despite the presence of large amounts of unsaturated 18:1 and 18:2 FA, only saturated acids are selected for elongation and decarboxylation. In this case, either the rapid incorporation of oleoyl-CoA into PhL occurs or the elongation enzymes are highly specific here and work only with saturated acyl-CoA [91].

Very little is known about the synthesis of hydroxy acids 20:1OH (leecarolic) and 20:2OH (auricolic). Currently, the transcript level of three *Lesquerella fendleri* genes: *FAH12* (bifunctional oleate-12-hydroxylase:desaturase), *KCS3* and *FEN1* (oleate-12-desaturase), has been determined, and the relationship between gene expression and the accumulation of very-long-chain hydroxy acids is discussed [92].

Modern genetic technologies and an increased understanding of the metabolic pathways of VLCFA synthesis have allowed the production of oil crops with a modified VLCFA profile. Very-long-chain polyunsaturated fatty acids, such as arachidonic acid (20:4), eicosapentaenoic acid (20:5) and docosahexaenoic acid (22:6), are valuable commodities that provide important human health benefits. Using transgenic technologies, it was shown to be possible to achieve an arachidonic acid level of up to 25% and eicosapentaenoic acid of up to 15% of the total fatty acids in *Brassica juncea* seeds. This also demonstrated the practical feasibility of the large-scale production of docosahexaenoic acid in oilseed crops [93].

TAG containing modified fatty acids with a functionality beyond those found in commercially grown oil seed crops can be used as feedstocks for the food industry. Over the years, advances have been made in transgenically engineering the production of various modified fatty acids in the model plant *Camelina sativa*. Using a set of heterologous desaturase and elongase genes, the authors achieved a significant accumulation of eicosapentaenoic (20:5) and docosahexaenoic (22:6) acids in the seed oil of the crop *Camelina sativa*. The achieved very-long-chain polyunsaturated FA levels are equivalent to those in fish oils [94]. The ease therefore of manipulating lipid compositions, combined with the ability to grow *Camelina sativa* in the field, make it an ideal platform to develop industrial lipids in transgenic oil seed crops [95].

## 6. The Synthesis of C_28–38_ VLCFA

In addition to the VLCFA found in plant tissues with a chain length up to C_26_ and, only in some cases, C_28_, VLCFA with a chain length of C_28–38_ are also often synthesized in plants, which can occur in cuticular waxes and suberin in the forms of esters with alcohols or in free form. VLCFA often serve as precursors of n-alkanes, ketones or alcohols, which are formed from them very quickly, leaving no initial compounds in the tissues [96]. Therefore, the issues of cloning and characteristics of the genes and corresponding enzymes involved in the biosynthesis of C_28–38_ VLCFA, as well as cuticular waxes and suberin, are often considered together. The initial VLCFA molecules enter the biosynthetic pathways of waxes and suberin through acyl reduction and decarbonylation and then are converted into a number of constituent components of these compounds [97]. The process of wax transfer to the epidermal surface began to be studied after the discovery of CER5, an ATP-binding cassette transporter (ABC) involved in the export of cuticular waxes to the stem surface [19].

**Figure 4:**
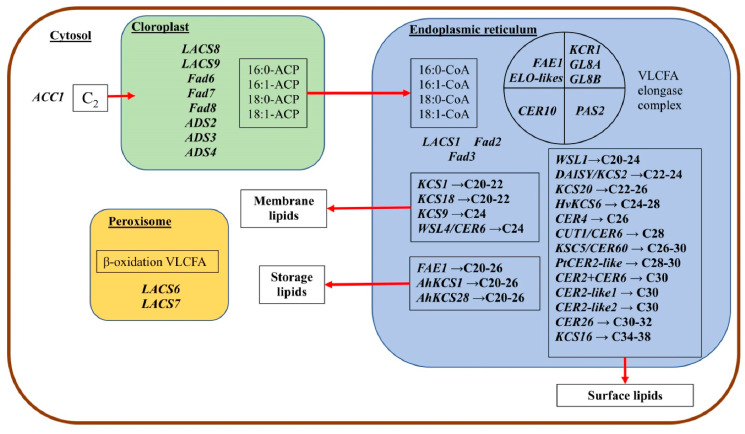
Genes encoding the elongation and desaturation enzymes involved in the synthesis of VLCFA and their derivatives. The *ACC1* gene encodes cytosolic acetyl-CoA carboxylase, which synthesizes malonyl-CoA, used for the formation of regular-chain FA in chloroplasts and for the elongation of VLCFA in the endoplasmic reticulum (ER). Elongation of the VLCFA occurs with the help of the sequential operation of four enzymes of the elongase complex. Two gene families: *FAE1* and *ELO-likes* encode β-ketoacyl-CoA synthase (KCS). The *KCR1*, *GL8A* and *GL8B* genes encode β-ketoacyl-CoA reductase (KCR); *PAS2* encodes β-hydroxyacyl-CoA dehydratase (HCD) and *CER10*–enoyl-CoA reductase (ECR). For the synthesis of VLCFA of different lengths, which are included in the membrane, surface and storage lipids, some specific genes are needed. The *Fad2*, *Fad3*, *Fad6*, *Fad7*, *Fad8*, *ADS2*, *ADS3* and *ADS4* genes encode the desaturases localized in the ER and chloroplasts. The *LACS* gene family encodes numerous long-chain acyl-CoA synthases that are involved in several metabolic pathways, starting from FA activation, including the biosynthesis of PL, triacylglycerols (TAG), jasmonate and β-oxidation of VLCFA in peroxisomes.

The synthesis of VLCFA for the subsequent formation of special apoplastic lipids requires a unique biochemical mechanism. It is accepted that the condensing enzymes (KCS) not only catalyze the first reaction in FA elongation but also determine the length of FA chains produced by the whole elongation complex. However, none of these enzymes can efficiently synthesize C_28–38_ VLCFA, which are precursors of a number of components of the most common cuticular waxes and pollen coats of most plant species [34]. It is recognized that the formation of saturated C_28–38_ VLCFA and their further transformation into a number of aliphatic compounds included in cuticle wax and suberin require the coordinated activity of a large number of enzymes. Thus, 282 candidate genes have been found that may play a role in the synthesis, regulation and transport of waxes [98]. Some genes involved in the synthesis of VLCFA are shown in Figure 4.

In addition to the four core enzymes forming the FAE complex, other proteins were shown to play important functions, including CER2-LIKE proteins, a group of five proteins homologous to BAHD acyltransferases. The proteins of the CER2-like family are known as the main components of FA elongase in Arabidopsis, maize and rice, which perform a specific function in the synthesis of C_28–34_ fatty acyl-CoA serving as precursors for several components of cuticular waxes [34,99,100]. In the Arabidopsis *cer2* mutant, the presence of a specific deficiency in the cuticular waxes of lipid chains longer than C_28_ was shown. It has also been shown that, with the heterologous expression of *CER2* in yeast, it can change the length of the acyl chain produced by the condensing enzyme from C_28_ to C_30_. *CER2* homologs: *CER2-like1* and *CER2-like2* were found to have a similar effect on the substrate specificity of condensing enzymes. The *CER2-like3* and *CER2-like4* genes homologous to BAHD acyltransferase are also described [101]. Thus, each of the CER2-like proteins induces changes in the length of the acyl chain that lead to the synthesis of the required cuticular waxes and pollen coats [34,100].

A number of genes from the *CER2-like* family have been characterized in Arabidopsis: *CER26* and *CER26-like*. It was found that the *cer26* mutation affects the production of wax components longer than C_30_. The analysis of the acetyl-CoA pool in the corresponding transgenic lines confirmed that the inactivation of this gene specifically affects the process of FA elongation beyond 26 C atoms. In addition, the expression of *CER26* in transgenic plants has shown that *CER26* promotes the elongation of C_≥30_ VLCFA with high tissue and substrate specificity [100].

The information on the functional conservation of this gene family in other plant species is insufficient. Two genes of the *CER2-like* families were studied: *NnCER2* and *NnCER2-like* from the sacred lotus (*Nelumbo nucifera*). Corresponding proteins were localized in the ER and nucleus. The overexpression of these genes in Arabidopsis led to a change in the structures of the cuticular waxes in the stems of inflorescences, and it was associated with an increase in the contents of components with a C_30–34_ chain length in waxes [99]. In *Populus trichocarpa*, this family consists of seven paralogous genes (*PtCER2-like1-7*). Five of them were heterologously expressed in yeast, and it was shown that *PtCER2-like* differentially altered the accumulation of C_28–30_ VLCFA when expressed in the presence of condensing enzyme CER6. Among them, *PtCER2-like5* produced the highest level of C_28_ in yeast [102]. The *Gl2* and *Gl2*-*like* genes in maize are also members of the BAHD acyltransferase family with close sequence similarity to the Arabidopsis *CER2* gene. These genes have a special effect on the synthesis of acyl lipids with C_32_ and more [103].

It has been shown that the *DAISY/KCS2* gene is also involved in the biosynthesis of suberin and the formation of protective layers in tissues. The Arabidopsis mutant *daisy* [4] is defective in the condensing enzyme of FA elongase. The roots of this mutant have growth disorders, and the content of C_22–24_ VLCFA decreases in suberin, while, compared to wild-type suberin, C_16–20_ FA accumulate. This indicates that DAISY functions as a docosanoic acid synthase. The transcription of the *DAISY/KCS2* gene is activated under the influence of NaCI, polyethylene glycol and an injury [61].

It is known that the biosynthesis of cuticular waxes in Arabidopsis requires the *CUT1/KCS6/CER6* gene encoding one of the condensing enzymes of VLCFA [14]. Suppression of the *CUT1* function in transgenic Arabidopsis plants resulted in an almost complete absence of the wax coating of plant stems. Waxy components with a C_24_ chain length predominate in such plants, suggesting that *CUT1* is necessary for the elongation of C_24_ VLCFA [104].

Mutations in Arabidopsis *CER6* genes eliminate lipids containing VLCFA from the cuticle surface and, in some cases, from the pollen coat, disrupting the interaction with the stigma and causing sterility. *CUT1/KCS6/CER6* was cloned, and the wild-type copy was shown to correct the *cer6-2* defect. The amino acid sequence of *CER6* is similar to that of the condensing enzymes, which is compatible with its role in the synthesis of epicuticular waxes and lipids of the pollen coat with a length of more than 28 C atoms [105,106]. It is known that *CER6* can only allow VLCFA chain elongation to C_28_, whereas the combined action of *CER6* and *CER2* leads to the synthesis of C_30_-VLCFA [6]. The *CER60/KCS5* gene has a high degree of nucleotide and amino acid homology with *CER6* [19,105]. The *SER60/KCS5* gene is involved in the synthesis of C_26–30_ VLCFA, as well as VLCFA C_20:1_ [6].

β-ketoacyl-CoA synthase CER6 is required for the biosynthesis of VLCFA with a chain length above C_28_ in tomato fruits. The mutation in the *SlCER6* gene impairs fertility and flower morphology. SlCER6 contributes to sexual reproduction and tomato flower development. The loss of SlCER6 function provokes a decrease in the amount of n- and iso-alkanes with a chain length of C_27_ and more and anteiso-alkanes with C_28_ or more in flower cuticular waxes, which affects the sexual reproduction and development of tomato flowers but does not affect the ultrastructure of the flower cuticle and the content of cutin. Wax deficiency was the reason for the fusion of flowers observed in tomatoes. The *slcer6* mutant has male sterility caused by obstructed pollen dispersion and abnormal tapet development [107]. The specificity of the SlCER6 substrate may be related to the elongation of not only linear but also branched long-chain FA, leading to the formation of corresponding alkanes. Thus, the *SlCER6* gene is important for regulating microgametogenesis in tomatoes [107].

The *WSL4* gene encoding KCS has been isolated from rice (*Oryza sativa*), whose homolog is *CER6/KCS6* in Arabidopsis and which is involved in the elongation of VLCFA beyond C_22_. *WSL4* overexpression increased the content of cuticular waxes in rice leaves. The *wsl-2* and *wsl-1* mutants had a markedly reduced content of wax components with lengths greater than C_28_ and C_30_, respectively [108].

One of several known genes involved in the biosynthesis of suberin has been isolated from potatoes (*Solanum tuberosum*). This gene, named *StKCS6*, was stably suppressed in potatoes by RNA interference. The downregulation of *StKCS6* affects the chain length distribution of VLCFA and their derivatives found in suberic polyester and peridermal wax. All compounds with a chain length of C_≥28_ were represented significantly less than aliphatic chains with C_≤26_ that accumulated. Thus, *StKCS6* is predominantly involved in the formation of suberic and waxy lipid monomers with a chain length of C_≥28_ [109].

It is known that the *LACS1/CER8* gene is involved in cuticular lipid synthesis [54]. LACS1, localized in the ER, is one of the nine Arabidopsis long-chain acyl-CoA synthetases that preferably modifies VLCFA for the synthesis of cuticular waxes, as well as C_16_ FA for cutin synthesis. It has been shown that LACS1 has synthetase activity for C_20–30_ VLCFA, with the highest activity for C_30_ VLCFA [23]. The epidermal-specific LACS2 synthetase also forms the Arabidopsis leaf cuticle and suggests that specific acyl-CoA pools may be involved in cutin biosynthesis [19]. Synthetases LACS1 and LACS2 have overlapping functions in the synthesis of both waxes and cutin. In addition, the activity of the LACS1 and LACS2 proteins in cuticle biosynthesis associated with the transmembrane and intracellular distribution of lipids intended for export to the cuticle was shown [110].

It is known that acyl-CoA VLCFA are converted into primary alcohols and wax esters by the reduction of acyl with the participation of the *CER4* and *WSD1* genes or they enter the alkane-forming pathway for the synthesis of aldehydes, alkanes, secondary alcohols and ketones with the participation of *CER1*, *CER3*, *CYTB5-b* and *MAH1* [99]. Characterization of the Arabidopsis *CER4* wax biosynthesis gene was performed. The *cer4* mutant of this plant shows a significant decrease in the content of stem primary alcohols and wax esters, as well as a slight increase in the content of aldehydes, alkanes, secondary alcohols and ketones. It is suggested that *CER4* encodes the alcohol-forming fatty acyl-CoA reductase (FAR), which is localized in the ER. Eight reductase-like genes have been identified in Arabidopsis. Molecular characterization of the *CER4* alleles and genomic complementation showed that one of these genes encodes the FAR required for the synthesis of cuticular waxes. The expression of *CER4* in yeast resulted in the accumulation of the C_24:0_ and C_26:0_ primary alcohols. *CER4* was expressed in the leaves, stems, flowers and the elongation zone of young roots. Thus, the *CER4* gene plays an important role in the biosynthesis of cuticular waxes. The alcohol-forming role FAR is specific for VLCFA and is responsible for the synthesis of primary alcohol in the epidermal cells of both aboveground tissues and roots [111].

n-6 monounsaturated primary alcohols (C_26_, C_28_ and C_30_) were found in the cuticle waxes of the Arabidopsis inflorescence stem. The Arabidopsis mutant *cer17* was completely devoid of these alcohols, and *CER17* was found to encode acyl-CoA desaturase 4 (ADS4). Studies of the Arabidopsis *cer4* mutant and yeast expressing the *CER4* gene in different ways and *CER17/ADS4* showed the important role of *CER4* in the synthesis of these monounsaturated alcohols. It has been shown that *CER17/ADS4* controls the desaturation of n-6 long-chain acyl-CoA in both the distal and basal inflorescence stems and regulates the amount of cutin monomer in them [99].

Arabidopsis orthologs of *KCS1*, designated as *BnKCS1-1* and *BnKCS1-2*, were identified in *Brassica napus*. The overexpression of *BnKCS1-1* and *BnKCS1-2* in the leaves of transgenic *B. napus* resulted in a significant increase in the amount of cuticular waxes. These changes were accompanied by higher levels of aldehydes (C_29–30_); alkanes (C_27_, C_29_ and C_31_) and secondary alcohols (C_28–29_) [112].

The relatively high content of C_35_ and C_37_ alkanes in Arabidopsis leaf trichomes suggests a difference in the mechanisms of the aliphatic chain elongation in individual epidermal tissues. These alkanes are formed, possibly, with the participation of KCS16, an enzyme expressed in the leaves, rosettes, flowers and anthers of Arabidopsis, with the localization of these proteins in the ER. The loss of *KCS16* function in mutants led to the fact that cuticular waxes in young leaves and isolated leaf trichomes were depleted in the C_35_ and C_37_ alkanes and alkenes, whereas the expression of Arabidopsis *KCS16* [6] in yeast and overexpression in Arabidopsis resulted in the accumulation of compounds formed from C_36–38_ VLCFA. Thus, KCS16 is the only enzyme that catalyzes the elongation of acyl-CoA from C_34_ to C_38_ in Arabidopsis leaf trichomes [1].

## 7. The Regulation of VLCFA Synthesis

**Figure 5:**
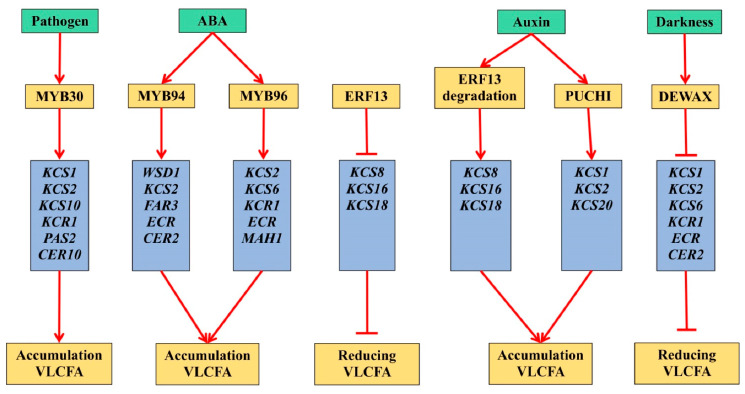
The regulation of the synthesis of VLCFA in plant cells. The diagram shows four transcription factors (MYB30, MYB94, MYB96 and PUCHI) that positively regulate VLCFA synthesis, and two (ERF13 and DEWAX) regulate it negatively. Pathogen-induced MYB30, ABA-dependent MYB94, MYB96 and auxin-dependent PUCHI activate the expression of genes for VLCFA biosynthesis, which leads to the accumulation of VLCFA in the cell. ERF13 and darkness-induced DEWAX suppress the expression of elongase complex genes and reduce the synthesis of VLCFA in the cell. Auxin-dependent degradation of the ERF13 results in the induction of *KCS8*, *KCS16* and *KCS18* gene expression and the accumulation of VLCFA in the cell. Arrows indicate a positive regulation. Lines ending with a bar indicate a negative regulation.

Currently, the regulation of VLCFA synthesis is studied very poorly. Transcription factor MYB30 is described in Arabidopsis, the synthesis of which is induced by the attack of pathogens (Figure 5). Studying transgenic plants in which MYB30 was overexpressed or suppressed, it was found that MYB30 controlled the expression of major genes of the elongase complex in Arabidopsis [113].

It is also known that, in Arabidopsis cells, MYB96 protein acts as an ABA-dependent transcription factor that activates the expression of genes for the VLCFA-condensing enzymes involved in cuticular wax biosynthesis. The *myb96-1d* mutant had a cuticular wax accumulation, whereas, in the *myb96-1* mutant, the reverse process was observed [114]. The Arabidopsis *MYB96* gene was overexpressed in the camelina plant (*Camelina sativa* L.) under the control of the *CaMV35S* promoter. The transgenic plants showed normal growth and development and increased the resistance to drought. At the same time, the levels of the alkanes and primary alcohols increased markedly. The identification of the cuticular wax biosynthesis genes made it possible to determine that the expression levels of *CsKCS2*, *CsKCS6*, *CsKCR1-1*, *CsKCR1-2*, *CsECR* and *CsMAH1* were two to seven times higher in the leaves of transgenic plants compared to the original ones [115]. Another ABA-dependent transcription factor, MYB94, was found to activate the expression of the *WSD1*, *KCS2/DAISY*, *CER2*, *FAR3* and *ECR* genes by binding directly to their promoters and enhancing the biosynthesis of cuticular waxes in Arabidopsis [116]. The transcription factor FDL1/MYB94, which is involved in cuticle formation, was found in maize (*Zea mays* L.) [35].

It has been shown that auxin-dependent transcription factors PUCHI and ERF13 regulate different groups of *KCS* genes involved in the VLCFA synthesis necessary for correct lateral roots development. The transcription factor PUCHI regulates the expression of *KCS1*, *KCS2* and *KCS20*, and ERF13 controls the *KCS8*, *KCS16* and *KCS18* genes [117]. Another transcription factor, DEWAX, belonging to the AP2/ERF-type transcription factors, has been described as a negative regulator of cuticular wax synthesis. In the dark, DEWAX suppresses the expression of the genes responsible for the elongation of VLCFA, such as *KCS1*, *KCS2*, *KCS6*, *KCR1*, *ECR* and *CER2* [118].

## 8. Conclusions

Despite the large amount of biochemical and genetic studies carried out, our knowledge of the all stages of C_20–38_ VLCFA biosynthesis in plants is still quite limited. Summing up the information obtained, it can be confirmed by the example of Arabidopsis that individual KCS are capable of producing VLCFA of different lengths: C_20–22_ (KCS1 and KCS18); C_22–24_ (KCS2, KCS20 and KCS9); C_24_ (KCS17); C_24–28_ (KCS5 and KCS6) and VLCFA up to C_38_ (KCS16 + KCS6 + CER2-like) [7].

In previous works, the results of the actions of various *KCS* genes, leading to the formation of either only one VLCFA or a whole group of these compounds, were presented. Sometimes, the same *KCS* genes are involved in the synthesis of not only saturated but also monounsaturated VLCFA [6]. These facts require further clarification and explanation. Experimental data on the participation of *KCS* genes in the synthesis of individual VLCFA or their groups in the model plant Arabidopsis should be confirmed by data obtained on other plant species. Only in this case is it possible to draw generalized conclusions for all plant tissues.

Recently, VLCFA esters with acyl-CoA have been recognized as key intermediates in numerous processes of lipid metabolism in eukaryotic tissues, as well as important compounds for the regulation of cell signaling. The intracellular concentration of these esters is controlled mainly by de novo FA synthesis, the activity of LACS synthetase and acyl-CoA thioesterase, the rate of β-oxidation and, finally, the concentration of lipid-binding proteins in contact with long-chain acyl-CoA esters. First of all, these conclusions relate to animal tissues, but very likely, they also apply to plant tissues [30].

It is assumed that intracellular transport and regulatory properties of acyl-CoA esters are coordinated by the acyl-CoA-binding domain, containing proteins. This domain is a highly conserved multigene family of intracellular lipid-binding proteins and is found in all eukaryotes. These proteins are involved in many intracellular processes, including the biosynthesis of FA and glycerolipids, β-oxidation, the differentiation and proliferation of cells and the regulation of the activity of many enzymes. The role of the acyl-CoA-binding domain in the regulation of these processes is still poorly understood; however, the data on their specific involvement in cellular signaling and lipid metabolism have been obtained. Among a number of such domains, ACBP is the most studied; this acetyl-CoA-binding protein is necessary for FA chain elongation and the synthesis of sphingolipids in eukaryotes [30]. In Arabidopsis, ACBP is encoded by a family of six genes, and one of them (*ACBP3*) participates in the plant’s response to hypoxia, modulating the metabolism of VLCFA [119].

Compounds serving as carriers of various intermediate products (for example, carnitine) can affect the final results of the biosynthesis of individual VLCFA. In this case, the decrease in the yield of any FA may be due not to the blockade of the synthesis of intermediates but to the impossibility of their transport to the site of synthesis of the required FA [32]. The final result may also be influenced by the activity of enzymes that are not directly involved in the synthesis of FA, such as carnitine acyltransferase, glycerokinase, diacylglyceroacyltransferase, etc.

Probably, the functioning of each synthesis block can be determined not by individual genes but by dozens of structural and regulatory genes combined into complex gene networks that alone provide coordinated regulation of their expression [32]. These data show that, currently, with rare exceptions, no special enzymes and genes responsible for the synthesis of VLCFA have been found in plants that differ from those that create FA of regular lengths; this applies to both enzymes of the elongation cycle of acid molecules and desaturases. At the same time, new genes are constantly being discovered that are involved in the control of one or another enzyme for the synthesis of regular FA (mainly, their elongation), simultaneously associated with both the synthesis of VLCFA and with any process of plant growth and development. In the future, it will be necessary to establish the structure, stoichiometry and organization of the VLCFA elongase complex in the ER in more detail. It can be stated that a number of questions that arise in the study of VLCFA biosynthesis have not yet been answered.

In the paper of Batsale et al. [7], numerous KCS genes that make up whole series were mentioned—for example, a list of KCS 1, 2, 4, 8, 11, 20 and 21 was given, and an opinion about the high functional redundancy of the number of these genes was expressed. It seems to us that nature cannot have redundant and unnecessary genes, and biosynthesis is not so much influenced by individual genes as by their totality in gene networks. The study of these gene networks is still in the initial stage. Facts are already being discovered when two genes in a pair give better results in the biosynthesis of VLCFA than each separately. Probably, further studies will find the complete gene networks that will show even better results in the biosynthesis of both VLCFA and their derivatives in cuticular waxes and suberin. Modern genetic technologies, such as CRISPR-Cas9, could help address the exact function of each gene involved in VLCFA synthesis through the generation of single and multiple mutants.

The studies of VLCFA synthesis are of great importance for the food industry in conditions of anthropogenic climate warming. The location on the surface of terrestrial plant tissues makes the cuticle, the components of which are VLCFA, an important player in protecting plants from unfavorable environmental factors, such as drought and pathogen attacks. Overexpression of the *BnKCS1-1* and *BnKCS1-2* genes led to an increase in the synthesis of surface waxes and reduced water loss in *Brassica napus* under drought conditions [112]. The results obtained indicate that the components of cuticular waxes dependent on the synthesis of VLCFA may be a key factor determining the germination of fungal spores on the surfaces of plants [66]. The isolation of the enzymes involved in the synthesis of VLCFA from different plants and an increased understanding of the metabolic fluxes when developing seeds have allowed the production of high levels of unusual lipids in transgenic oil seed crops such as *Camelina sativa* [95]. The use of transgenic engineering will allow the creation of new varieties of oil seed crops and agricultural plants with a modified composition of cuticular waxes that are more resistant to drought and pathogen attacks.

## Data Availability

The data are contained within the article.
